# Systemic inflammation increases mast cell numbers in coronary arteries in patients who died from myocardial infarction

**DOI:** 10.1016/j.jmccpl.2026.100870

**Published:** 2026-09-24

**Authors:** M.D. Fiet, W.W. Fuijkschot, L. Woudstra, R. van der Linden, D. Beskers, I.P.A. Zethof, L.F.H.J. Robbers, S. Simsek, P.A.J. Krijnen, H.W.M. Niessen, Y.M. Smulders

**Affiliations:** a Department of Pathology, Amsterdam University Medical Centers, Amsterdam, the Netherlands; b Amsterdam Cardiovascular Sciences, Amsterdam University Medical Centers, Amsterdam, the Netherlands; c Department of Cardiology, Amsterdam University Medical Centers, Amsterdam, the Netherlands; d Department of Internal Medicine/Endocrinology, Amsterdam University Medical Centers, Amsterdam, the Netherlands; e Department of Cardiac Surgery, Amsterdam University Medical Centers, Amsterdam, the Netherlands

**Keywords:** Atherosclerosis, Sepsis, Myocardial infarction, Inflammation, Human autopsy study

## Abstract

Systemic inflammation caused by infection peaks the risk of acute myocardial infarction (AMI), presumably by affecting atherosclerotic plaque (AP) stability. In this study, APs were examined in AMI patients with acute infection (AI).

Four groups of AMI patients with or without AI were compared to determine the effects of infection on both early (<6 h) and late (6 h–14 days) AMI: Early AMI, early AMI + AI, late AMI and late AMI + AI. Changes in the AP were determined by measuring stenosis (Elastica von Gieson), plaque stability (thickness of fibrotic cap and leukocytes presence) and intraplaque haemorrhage (GLUT-1 and Perls). Inflammation was determined by measuring leukocytes, macrophages, mast cells and neutrophilic granulocytes in the different regions of the coronary arteries: intima, media and adventitia.

AI did not affect coronary stenosis, plaque stability or intraplaque haemorrhage. Plaque instability increased in late AMI + AI compared to early AMI + AI (*p* = 0.0056). AI did not affect leukocyte-, macrophage- or neutrophil numbers in the coronary artery wall. However, increased mast cell densities were found in the intima and media in early AMI + AI patients (*p* = 0.022; *p* < 0.0001 respectively), while mast cells decreased significantly in the intima late AMI + AI patients (*p* < 0.0001), and non-significantly in the media (*p* = 0.0609).

Therefore we conclude that, in contrast to other inflammatory cell types, mast cell influx into the coronary artery wall is increased in patients with AI with early AMI. As mast cells produce vasoconstricting compounds, this may contribute to the increased risk of AMI in patients with AI.

## Highlights

• No gram-positive bacteria were present in the atherosclerotic plaque in AMI patients.
• Acute infection recruits mast cells to the coronary artery intima and media.
• An increase of inflammation is seen in the coronary arteries in late stage AMI.
• Intima, media and adventitia have different densities of inflammatory cells.

## Graphical abstract

**Figure:**
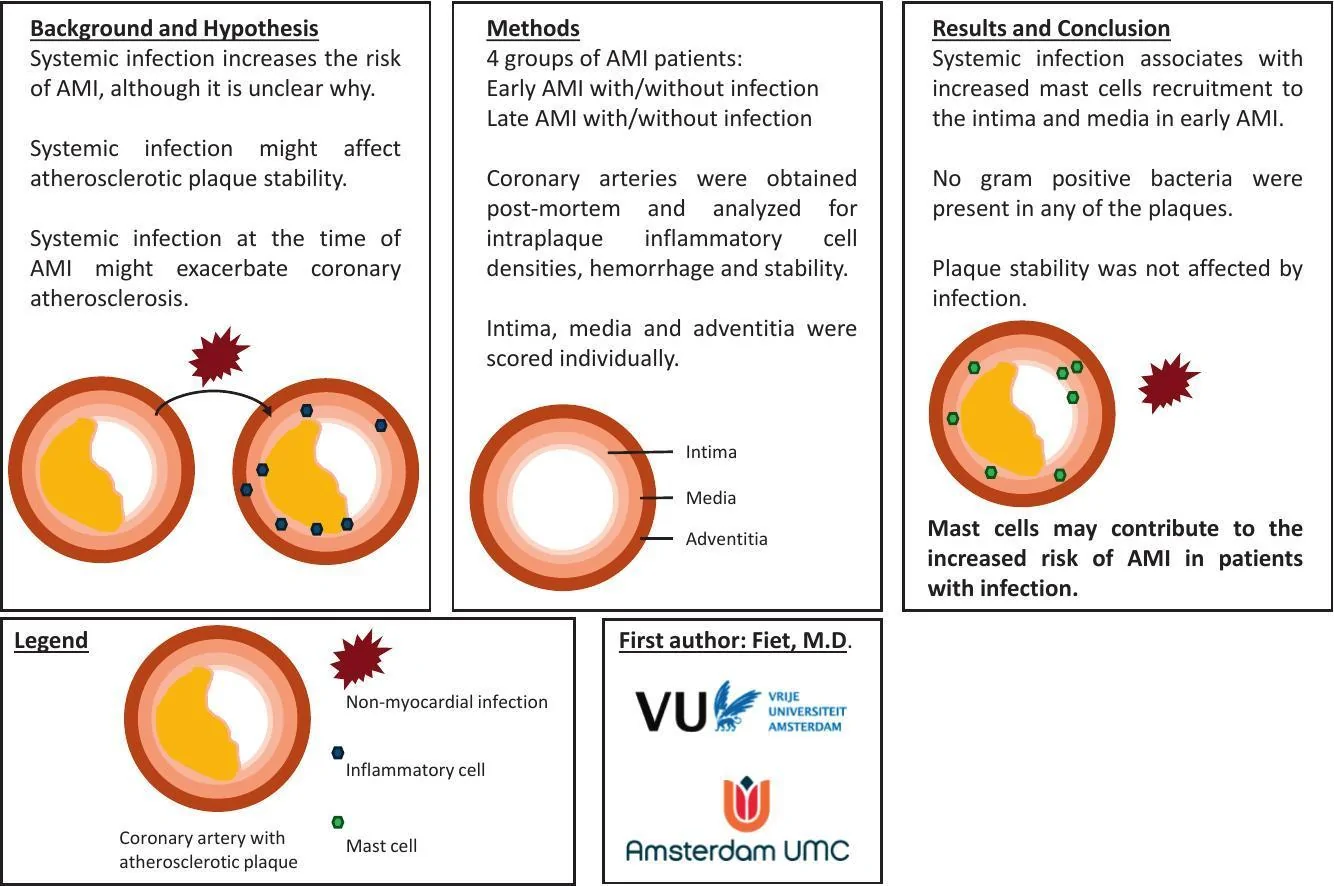

## 1 Introduction

Observational epidemiological studies show that cardiovascular events can be triggered by clinical conditions that are associated with systemic inflammation [1], [2], [3], [4]. Respiratory tract infection, for instance, raises the early (2–14 days after onset of symptoms) [3], [4] risk of acute myocardial infarction (AMI) approximately fivefold [4], and the risk of stroke eightfold [1], [4].

The causal mechanisms behind inflammation-induced cardiovascular event risk remain elusive, but its acuteness suggests an effect on atherosclerotic plaque stability [5], [6], [7]. Whether plaque stability is indeed affected, and by which mechanisms, is unclear. It is known that intra-plaque inflammation plays an important role in the progression and destabilization of atherosclerotic plaques [8], [9], [10], [11]. It is thus worth investigating whether acute inflammation has short-term effects on plaque inflammation.

Several studies previously addressed the effect of acute inflammation on atherosclerosis, but most focused on intermediate to long-term effects. For example, plaque area was increased in Apo E −/− mice 16 weeks after induction of systemic inflammation [12]. Plaque progression can also be amplified by infection as seen in a murine model of abdominal sepsis with increased macrophage infiltration into plaque atheroma [13], and urinary tract infection, which released bacterial endotoxins that elicited local inflammation in plaque atheroma [14]. Whether acute systemic inflammation induces similar pro-inflammatory intra-plaque changes in humans has, however, not been studied to date.

Given the short-term effects of systemic inflammation on cardiovascular event risk, we hypothesized that an acute episode of infection rapidly enhances intra-plaque inflammation. In the coronary arteries such changes could act in two ways: First, systemic inflammation could contribute to the occurrence of a primary AMI. Second, systemic inflammation could enhance the pro-inflammatory plaque changes that we know are evoked by recent AMI itself, thus bearing relevance for patients who have an infection in the early post-AMI period [15], [16]. We decided that both hypotheses could be addressed by analysing inflammatory cell content in atherosclerotic plaques in coronary arteries of patients who died at different time intervals after AMI with or without infection.

## 2 Methods

### 2.1 Patient selection and tissue acquisition

The study included deceased AMI patients referred to the Department of Pathology for clinical autopsy, which was performed within 24 h after death. Relatives of patients provided written consent for the use of autopsy material for scientific research. All patients showed macroscopic evidence of recent AMI, as identified by lactate dehydrogenase (LDH) decolouration of myocardial tissue. The diagnosis of STEMI was made using an ECG recording in living patients at the cardiology department. No ECG was available for patients that died out of the hospital. Patients diagnosed with chronic inflammatory disease (e.g. systemic lupus erythematosus or rheumatoid arthritis), recent surgery (<6 months) and metastatic disease were excluded, since these conditions could influence inflammatory status [2], [17], [18], [19], [20].

Patients with acute infection (AI) were selected based on clinically and autopsy proven sepsis or severe infection at time of death and compared to patients with no signs of infection (no-AI). In case of pneumonia, no additional staining was performed on the lung tissue to determine the infectious agent.

For this study, the AMI patients were subdivided according to infarct duration, e.g. patients with very early AMI who died within 6 h after AMI onset (designated ‘Early-stage AMI’) and in whom infarct-induced effects on atherosclerosis did not have sufficient time to establish, and patients with mid-term to late AMI who died within 6 h to approximately 14 days after AMI (designated ‘Late-stage AMI’). For this, myocardial tissue specimens were obtained from the centre of the LDH-decolourized infarct area, and formalin-fixed and embedded in paraffin for (immuno)histochemical analyses.

The infarct duration was based on microscopic criteria: the early-stage AMI phase patients showed macroscopic LDH decolouration, but no microscopic evidence yet of extravasation of neutrophilic granulocytes in the infarcted myocardium (Phase 1 AMI patients); the late-stage AMI patients showed macroscopic LDH decolouration and microscopic evidence of either cardiomyocyte necrosis combined with extravasation of neutrophilic granulocytes (Phase 2 AMI patients) or granulation tissue formation with or without cell-rich early fibrotic changes [21] (Phase 3 AMI patients) [22], [23], [24], [25]. Groups were as follows: Early stage AMI (*N* = 12), late stage AMI (*N* = 6), early stage AMI + AI (*N* = 5); late stage AMI + AI (*N* = 12).

In all patients, the left and right coronary arteries were excised from their root to the point where they enter the myocardium. Sections with the most severe stenosis were identified macroscopically and subsequently processed separately for histological and immunohistochemical analyses. Selected tissue cross-sections were fixed in 4% paraformaldehyde, decalcified in buffered formic acid, and embedded in paraffin.

### 2.2 Histochemistry and quantification atherosclerotic plaques and the myocardium

During autopsy, a routinely performed lactase dehydrogenase (LDH) staining was analysed to determine the location of cardiac injury. In short, fresh heart cross sections were incubated in nitro blue tetrazolium (NBT)-containing solution at 37 °C for 5–10 min. Areas where LDH has leaked from the damaged myocardium can be identified by the lack of LDH-mediated conversion of NBT to blue-coloured formazan (termed LDH decolouration). As not all patients received PCI with stenting, no additional histology was performed on stented portions of vessels. To determine presence of gram-positive bacteria in the plaque, a standard Gram-staining was performed on the coronary arteries of AI patients and subsequently scored by a pathologist.

Paraffin tissue sections (4 μm) of the coronary segments were stained with Elastica von Gieson (EVG) and haematoxylin-eosin (HE) for histological analyses according to standard protocols. In short, slides were first deparaffinized in xylene and dehydrated in graded ethanol (100–96-70%). Then slides were rinsed with tap water and incubated with Lawson's solution for one hour. Subsequently, the slides were stained with Haematoxylin (Klinipath #4085), blued with tap water, and counterstained with von Giesson solution for five minutes each. Finally, slides were air-dried and covered.

The degree of stenosis of each individual segment was determined based on EVG stained cross-section as percentage of luminal surface area compared to surface area defined by the internal elastic lamina. All atherosclerotic plaques were evaluated microscopically to verify the presence of a fibrous cap. Plaques were classified as unstable if the fibrous cap was thin and/or if multiple inflammatory cells reached up to the luminal endothelial layer of the plaque (based on anti-CD45 staining) [26], [27]. If neither of these characteristics were present, plaques were classified as stable. The culprit area (e.g. area with most stenosis) of the artery was studied to determine plaque erosion, plaque rupture and thrombus formation and the atherosclerotic plaques were described according to the Virmani classification (Type I: Macrophages, Type II: Lipids intracellular, Type III: Lipids extracellular, Type IV: Lipid core extracellular, Type V: Fibroatheroma; Type VI Complex consisting of erythrocytes, recent haemorrhage and/or thrombus).

To classify intraplaque haemorrhage (IPH) all coronary segments were stained for erythrocytes with the immunohistochemical GLUT-1 staining identifying fresh haemorrhage (see Immunohistochemistry section ahead) and for iron with the histochemical Perls staining identifying old haemorrhage. In short, staining consisted of a 30 min incubation of the slides in Perls solution, followed by a aqueous neutral red solution counter stain for 1 min. Using a microscope with 250 × magnification, fresh IPH was identified by the presence of >10 extravascular localized intact erythrocytes per plaque, while old IPH was identified by the presence of iron and/or erythrocyte fragments [26], [27]. Ongoing IPH was defined as both fresh and old haemorrhage [27].

### 2.3 Immunohistochemisty

Paraffin tissue sections (4 μm) of the myocardium and coronary segments were stained with antibodies detecting GLUT-1 (erythrocytes), CD45 (leukocytes), CD68 (macrophages), MPO (neutrophils), and tryptase (mast cells). For this, the sections were first deparaffinized, rehydrated and blocked for endogenous peroxidases by incubation in H_2_O_2_ (0.3%) diluted in methanol for 30 min. Antigen retrieval was performed by heat inactivation in sodium-citrate buffer (10 mM, pH 6.0) for slides to be stained for CD68, MPO and tryptase or in a Tris/EDTA buffer (pH 9.0) for slides to be stained with GLUT-1. Slides to be stained for CD45 required no antigen retrieval step. The slides were incubated with either rabbit-anti-human Glut-1 (1:100, Thermo Scientific RB-9052-P), mouse-anti-human CD45 (1:100, Dako M0701), rabbit-anti-human CD68 (1:400, Dako M0814), mouse-anti-human MPO (1:1500, Dako A0398) or mouse-anti-human Tryptase (1:500, Dako M7052) for 1 h at room temperature. The sections were then washed with phosphate-buffered saline and incubated with Real EnVision HRP α-mouse/rabbit (undiluted; Dako) for 30 min at room temperature. The staining was visualized using 3,3′-diaminobenzidine (0.1 mg/ml, 0.02% H2O2). Lastly, the sections were counterstained with haematoxylin, dehydrated, and covered. With each staining, a phosphate-buffered saline control and an isotype control were included. All these controls yielded negative results.

### 2.4 Quantification of immune cells and morphometric analysis

In all individual segments of each coronary artery the total surface area of the intima, media and adventitia were determined on the scanned slides using the Panoramic Desk scanner and analysed with Pannoramic Viewer 1.15.2 software (3DHistech, Budapest, Hungary). The surface area of adventitia was measured from the external elastic lamina until 100 μm into the tunica externa.

CD45+ leukocytes and CD68+ macrophage numbers were analysed with ImageJ 1.47 software (National Institutes of Health, USA) using a colour threshold to determine the surface area of positive anti-CD45 and anti-CD68 staining and dividing it by the surface areas of the relevant intima, media and adventitia. CD68 positive area was quantified only in the intima, since there were no areas of positive staining present in the media and adventitia. The numbers of extravascular mast cells and neutrophilic granulocytes were counted using a light microscope using a 25× magnification objective (Zeiss, Germany) and then calculated per mm^2^ of the surface areas of the intima, media and adventitia. To ensure validity of the scoring, a selection of the slides was scored by a second assessor and the mean score was taken. No scores differed more than 10% of each other.

### 2.5 Statistics

To evaluate differences in plaque area, plaque type, lesion number and inflammatory cells between groups, Kruskal-Wallistesting (non-parametric data) and Chi^2^-testing (non-parametric data) were used as appropriate. Data distribution was tested for normal distribution by Shapiro-Wilk testing. Statistical analyses were performed using GraphPad Prism version 10.6.1 for Windows, (GraphPad Software, La Jolla California USA). Results were considered statistically significant if the two-sided *p*-value was <0.05.

## 3 Results

### 3.1 Baseline characteristics

35 AMI patients with a total of 295 coronary slides were included and categorized into 4 groups based on infarct duration and the presence of acute infection. Groups were as follows: Early AMI (*N* = 12, *n* = 120), late AMI (*N* = 6, *n* = 45), early AMI + AI (*N* = 5, *n* = 31); late AMI + AI (*N* = 12, *n* = 99).

Most patients had either a Type V (Fibroatheroma) (Early AMI: 6, Late AMI: 1, Early AMI + AI 0; Late AMI + AI 2) or Type VI (Complex with erosion, thrombus and/or recent haemorrhage) atherosclerotic plaque (Early AMI: 5, Late AMI: 5, Early AMI + AI 4; Late AMI + AI 10). One patient (Early AMI + AI) had a Type III (Extracellular lipids) atherosclerotic plaque (Table 1). Based on the LDH staining, only 1 patient (Late AMI + AI) had localized subendocardial and patchy decolourisation, potentially indicative of hypoperfusion.

**Table 1:** Baseline characteristics per group.

Characteristic				
(N)	12	6	5	12
Early or late infarct	Early	Late	Early	Late
Acute infection	No	No	Yes	Yes
Sex				
Male (%)	11 (91,7%)	5 (83,3%)	4 (80%)	8 (66,7%)
Age				
Years (mean ± SD)	67,8 (±15)	61,7 (±11)	73,4 (±21)	74,9 (±10)
STEMI				
Yes/no/unknown	2/1/9	3/2/1	0/3/2	0/4/8
Type atherosclerotic plaque				
Type III	–	–	1/5	–
Type V	6/12	1/6	–	2/12
Type VI (recent haemorrhage)	5/12	4/6	2/5	3/12
Type VI (thrombus)	–	–	–	1/12
Type VI (thrombus + recent haemorrhage)	–	1/6	1/5	2/12
Type VI (rupture + recent haemorrhage)	–	–	–	2/12
Type VI (erosion + recent haemorrhage)	–	–	–	1/12
Type VI (erosion + thrombus + recent haemorrhage)	–	–	–	1/12
Unknown	1/12	–	1/5	–
Cause of acute systemic inflammation				
Pancreatitis	–	–	–	1/12
Pneumonia	–	–	3/5	3/12
+Arthritis/fasciitis necroticans	–	–	1/5	–
+Catecholamine myocarditis	–	–	–	1/12
+Urinary tract infection	–	–	–	1/12
Sepsis	–	–	1/5	–
+Endocarditis	–	–	–	1/12
+Infected femoropopliteal bypass	–	–	–	1/12
+Ischemic colitis	–	–	–	1/12
+Myocarditis	–	–	–	1/12
+Pneumoni/endocarditis/infected mitral valve prosthesis	–	–	–	1/12
+Pneumonia/peritonitis/myocarditis	–	–	–	1/12

Abbreviations: SD: standard deviation; STEMI: ST-elevated myocardial infarction.

Gram stainings of all atherosclerotic plaques were performed to assess putative gram-positive bacterial infection of the plaques. All atherosclerotic plaques were Gram-negative, indicating no presence of gram-positive bacteria in the atherosclerotic plaque (data not shown).

Known infectious agents, diagnosis of STEMI and comorbidities of patients with infections and/or sepsis are presented in Supplementary Table 1. Diagnosis of STEMI and comorbidities of patients without infection are presented in Supplementary Table 2. Validation of gram stainings is provided in Supplementary Fig. 1. Validation of the monoclonal antibodies using an isotype control is shown in Supplementary Fig. 2. Negative controls of polyclonal antibodies are shown in Supplementary Fig. 3.

### 3.2 Infarct-related area, stenosis, plaque stability and intra-plaque haemorrhage

In AMI patients, no significant differences between the non-AI and AI groups were observed for infarct area of the left ventricle (Fig. 1A and Supplementary Fig. 4A), stenosis (Fig. 1B), plaque stability (Fig. 1C, Supplementary Fig. 4B) and intra-plaque haemorrhage (Fig. 1D, Supplementary Fig. 4C + D). When comparing AMI patients, late stage AMI patients were characterized by a significantly higher degree of stenosis compared to early AMI patients (72,7% occlusion vs 63,5% occlusion) (*p* = 0,048) (Fig. 1B). However, no significant differences in stenosis were found between infarct-related (e.g. the artery with the most stenosis) and non-infarct related arteries in either of the 4 patient groups (data not shown).

**Fig. 1:**
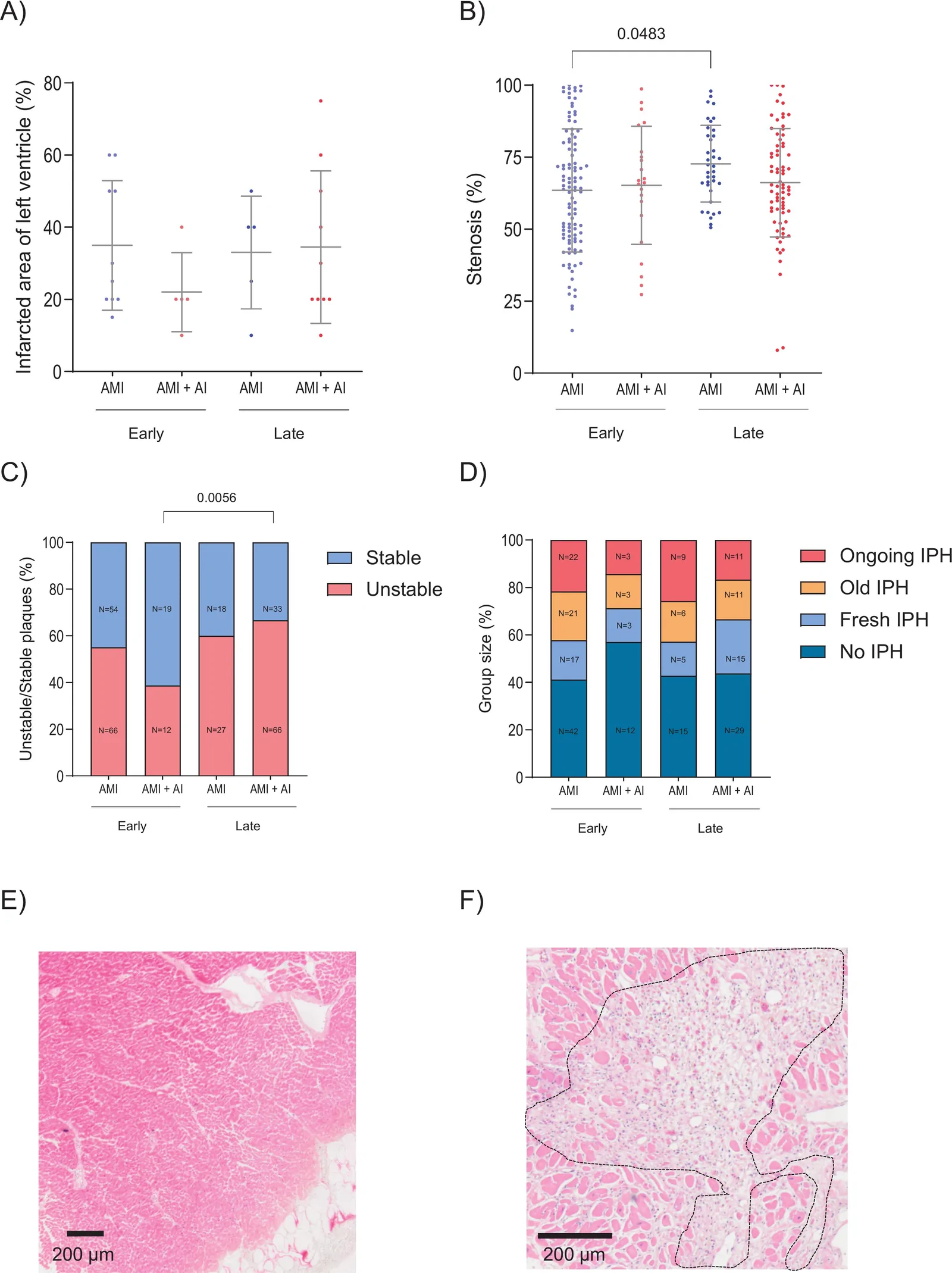
Infarcted area, stenosis and stability per group. A: Infarcted area as a percentage of the left ventricle divided by total area of the left ventricle. Samples consist of myocardial cross sections. Early AMI: *N* = 10, early AMI + AI: *N* = 5, Late AMI: *N* = 5 and Late AMI + AI: *N* = 10. Values were calculated via Kruskal-Wallis testing. Error bars represent mean and standard deviation, significant *p*-values (*p* < 0.05) are shown above the columns. B: Stenosis expressed as percentage of the surface area of the lumen divided by the surface area of the intima ending at the internal elastic lamina. Samples consist of atherosclerotic plaque cross sections. Percentage of stenosis of total lumen size: Early AMI: *n* = 103, early AMI + AI: *n* = 22, late AMI: *n* = 37 and late AMI + AI: *n* = 73. Values were calculated via Kruskal-Wallis testing. Error bars represent mean and standard deviation, significant *p*-values (*p* < 0.05) are shown above the columns. C: Percentages of unstable plaques and stable plaques. Percentages are given on the Y-axis, as well as an absolute number of plaques inside of the bar diagram. Samples consist of atherosclerotic plaque cross sections: Early AMI: *n* = 120, early AMI + AI: *n* = 45, late AMI: *n* = 31 and late AMI + AI: *n* = 99. Values were calculated via Chi^2^-testing. Significant *p*-values (*p* < 0.05) are shown above the columns. D: Percentages of plaques without and with either ongoing, fresh or old haemorrhage. Samples consist of atherosclerotic plaque cross sections. Percentages are given on the Y-axis, as well as an absolute number of plaque inside of the bar diagram: Early AMI: *n* = 102, early AMI + AI: *n* = 35, late AMI 6: *n* = 21 and late AMI + AI: *n* = 66. Statistical significance wase calculated via Ordinary two-way ANOVA testing. E: HE stain of an early stage AMI patient without signs of active inflammation/granulation tissue. F: HE stain of a late stage AMI patient with active granulation tissue.

Plaque stability was decreased in late stage AMI + AI patients (66% unstable plaques) compared to early stage AMI + AI patients (38.7% unstable plaques)(*p* = 0.0056) (Fig. 1C and Supplementary Fig. 5), while no significant differences in the occurrence of old and fresh intra-plaque haemorrhage were found between the groups (Fig. 1D). In early AMI patients no granulation was present (Fig. 1E), while in late AMI patients granulation tissue was present (Fig. 1F).

### 3.3 CD45+ leukocytes

In patients with early AMI, no significant differences between the non-AI and the AI group were found (Fig. 2A–C). In patients with late AMI, a significant decrease was found in the AI group compared with the non-AI group (*p* < 0.0001; *p* = 0.0139; *p* < 0.0001, for intima, media and adventitia, respectively; Fig. 2A–C).

**Fig. 2:**
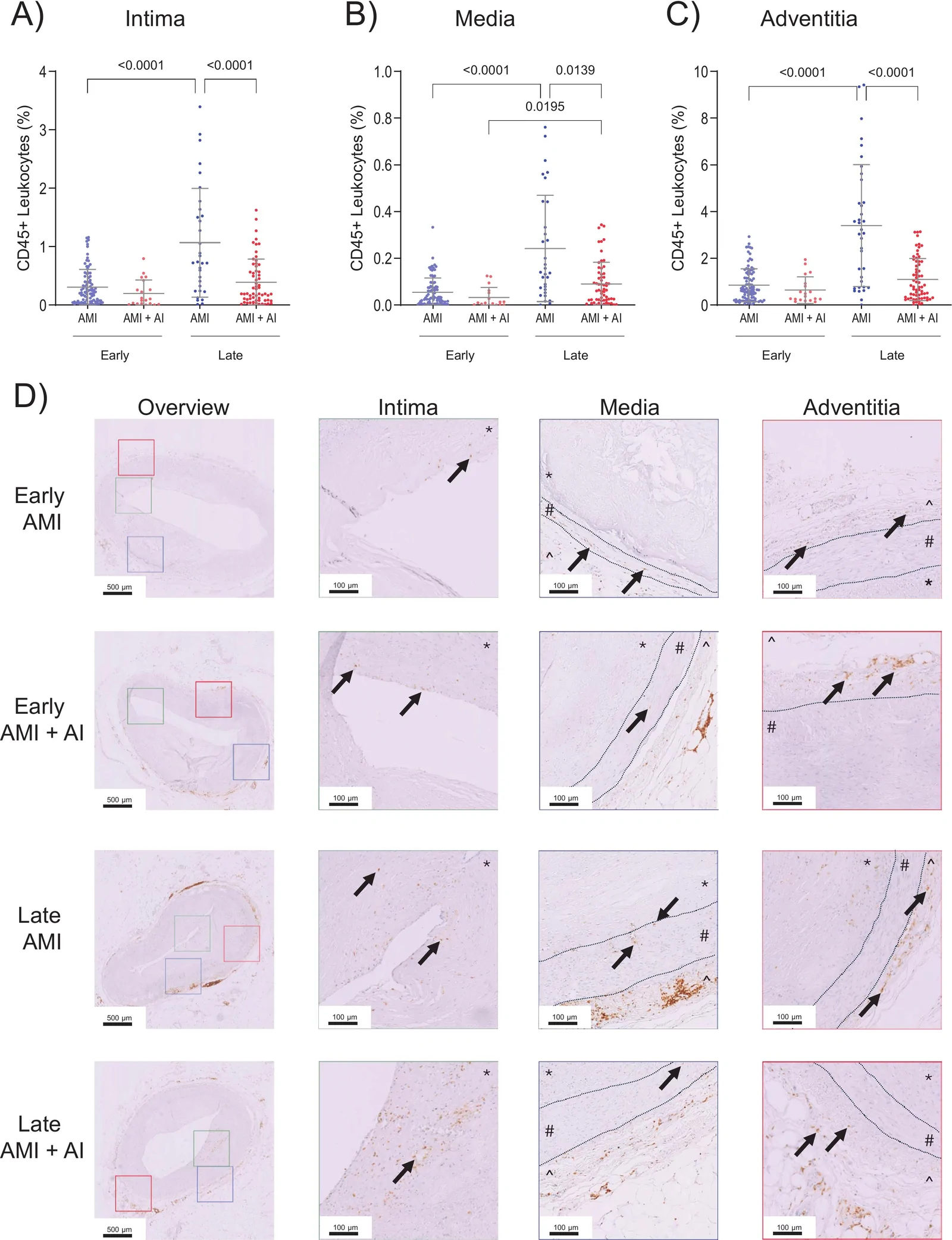
CD45+ leukocyte density per group. CD45 positive area of the total size of either the intima, media or adventitia of the coronary arteries. Samples consist of atherosclerotic plaque cross sections. A: Percentage of CD45 positive area of the total intima size: Early AMI: *n* = 89, early AMI + AI: *n* = 20, late AMI: *n* = 32 and late AMI + AI: *n* = 62. Values were calculated via ordinary one-way ANOVA testing. B: Percentage of CD45 positive area of the total media size: Early AMI: *n* = 86, early AMI + AI: *n* = 17, late AMI: *n* = 31 and late AMI + AI: *n* = 61. Values were calculated via Kruskal-Wallis testing. C: Percentage of CD45 positive area of the total adventitia size: Early AMI: *n* = 95, early AMI + AI: *n* = 19, late AMI: *n* = 36 and late AMI + AI: *n* = 66. Values were calculated via Kruskal-Wallis testing. D: CD45 staining for leukocytes (brown colourization, arrow points to an example of positive staining) for all patient groups, with higher magnification of the intima, media and adventitia. If multiple layers are present in the higher magnification photograph, a dotted line divides the layers and an “I” (Intima), “M” (Media) or “A” (Adventitia) denotes the area of interest All samples are independent and were processed once. All graphs values were calculated via Kruskal-Wallis testing. Error bars represent mean and standard deviation, significant *p*-values (*p* < 0.05) are shown above the columns. (For interpretation of the references to colour in this figure legend, the reader is referred to the web version of this article.)

Overall, CD45+ leukocytes densities were significantly higher in late AMI patients compared to the early AMI group in all layers (*p* < 0.0001, for intima, media and adventitia; Fig. 2A–C). CD45+ leukocytes of the late AMI + AI group were only increased in the media when compared to the early AMI + AI group (*p* = 0.0195, Fig. 2B). All values are shown in Table 2. Examples of the CD45 staining are presented in Fig. 2D.

**Table 2:** Results of various graphs

Characteristic	Intima				Media				Adventitia			
Early/late infarct	Early	Late	Early	Late	Early	Late	Early	Late	Early	Late	Early	Late
Acute infection	No	Yes	No	Yes	No	Yes	No	Yes	No	Yes	No	Yes
Leukocytes (% of area)	0.30 ± 0.31	0.20 ± 0.23	1.07 ± 0.93	0.39 ± 0.40	0.05 ± 0.06	0.03 ± 0.03	0.24 ± 0.23	0.09 ± 0.09	0.85 ± 0.71	0.64 ± 0.64	3.4 ± 2.6	1.1 ± 0.90
Macrophages (% of area)	1.53 ± 2.74	1.17 ± 1.83	2.79 ± 4.30	2.88 ± 4.82	–	–	–	–	–	–	–	–
Neutrophils (cells/mm^2^)	1.77 ± 2.81	2.64 ± 2.60	2.33 ± 2.35	1.17 ± 1.02	0.34 ± 0.61	1.21 ± 1.80	0.79 ± 1.25	0.71 ± 1.00	3.70 ± 3.31	5.43 ± 4.68	6.91 ± 6.26	4.61 ± 3.90
Mast cells (cells/mm^2^)	2.42 ± 3.28	3.84 ± 3.83	2.17 ± 1.47	3.41 ± 10.6	0.83 ± 2.48	4.21 ± 3.76	2.15 ± 9.44	5.13 ± 10.6	25.3 ± 25.4	17.8 ± 14.3	18.4 ± 10.5	17.5 ± 18.3

### 3.4 Macrophages

In patients with AMI, the density of intimal macrophages was similar between the AMI groups and AMI + AI groups. Overall, macrophage density was higher in late AMI patients versus early AMI patients (*p* = 0.0024). Likewise, late AMI + AI patients had an increase of macrophage density compared to early AMI + AI patients (*p* = 0.0239) (Fig. 3A). All values are shown in Table 2. Examples of the CD68 staining are presented in Fig. 3B.

**Fig. 3:**
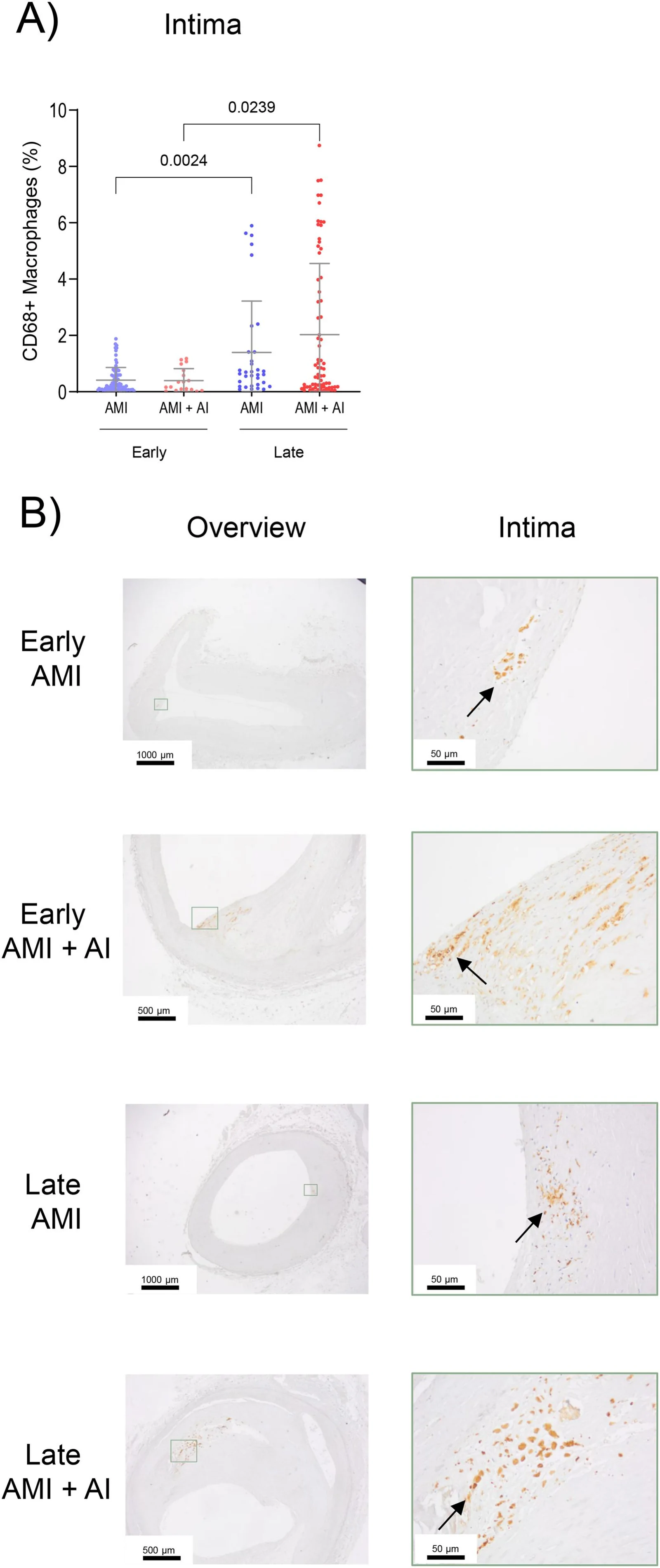
CD68+ macrophage density per group. A: Percentage of CD68 positive area of the total size of the intima. Samples consist of atherosclerotic plaque cross sections: Early AMI: *n* = 106, early AMI + AI: *n* = 23, late AMI: *n* = 39 and late AMI + AI: *n* = 76. Values were calculated via Kruskal-Wallis testing. B: CD68 staining for macrophages (brown colourization, arrow points to an example of positive staining) for all patient groups, with higher magnification of the intima, media and adventitia. All samples are independent and were processed once. All graphs values were calculated via Kruskal-Wallis testing. Error bars represent mean and standard deviation, significant *p*-values (*p* < 0.05) are shown above the columns. (For interpretation of the references to colour in this figure legend, the reader is referred to the web version of this article.)

### 3.5 Neutrophilic granulocytes

No significant differences in neutrophil density were found between early AMI patients and early AMI + AI patients, nor were differences found between late AMI patients and late AMI + AI patients (Fig. 4A–C). An increase of neutrophilic density was seen in late AMI patients compared to early AMI patients in the adventitia (*p* = 0.0259, Fig. 4C). All values are shown in Table 2. Examples of the MPO staining are presented in Fig. 4D.

**Fig. 4:**
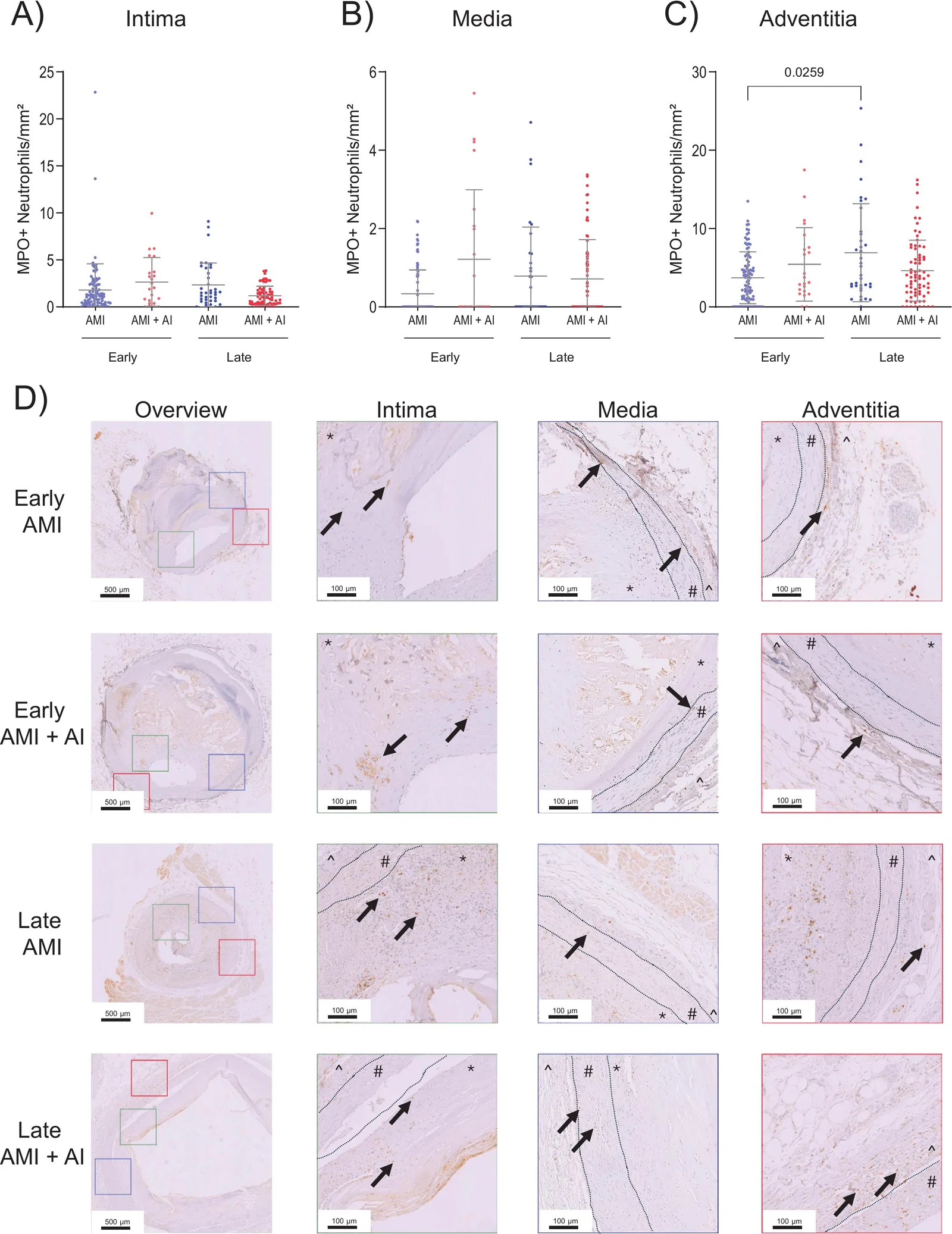
MPO+ neutrophil density per group. MPO+ neutrophils/mm^2^ in either the intima, media or adventitia of the coronary arteries. Samples consist of atherosclerotic plaque cross sections. A: Density in mm^2^ of MPO+ neutrophils in the total intima area: Early AMI: *n* = 98, early AMI + AI: *n* = 23, late AMI: *n* = 36 and late AMI + AI: *n* = 68. B: Density in mm^2^ of MPO+ neutrophils in the total media area: Early AMI: *n* = 94, early AMI + AI: *n* = 22, late AMI: *n* = 33 and late AMI + AI: *n* = 69. C: Density in mm^2^ of MPO+ neutrophils in the total adventitia area: Early AMI: *n* = 95, early AMI + AI: *n* = 22, late AMI: *n* = 36 and late AMI + AI: *n* = 72. D: MPO staining for neutrophils (brown colourization, arrow points to an example of positive staining) for all patient groups, with higher magnification of the intima, media and adventitia. If multiple layers are present in the higher magnification photograph, a dotted line divides the layers and an “I” (Intima), “M” (Media) or “A” (Adventitia) denotes the area of interest. All samples are independent and were processed once. All graphs values were calculated via Kruskal-Wallis testing. Error bars represent mean and standard deviation, significant *p*-values (*p* < 0.05) are shown above the columns. (For interpretation of the references to colour in this figure legend, the reader is referred to the web version of this article.)

### 3.6 Mast cells

In early AMI patients, higher counts of mast cells are present in the intima and media of AMI + AI patients than in the AMI patient group (*p* = 0.0222 and *p* < 0.001, respectively), but not in the adventitia (Fig. 5A–C). In contrast, late AMI + AI patients had a significant decrease in mast cell density in the intima (Fig. 5A, *p* < 0.001) and a non-significant decrease in the media (Fig. 5B, *p* = 0.0609) compared to the late AMI group. Interestingly, there was a significant increase of mast cells in late AMI-patients compared to early AMI-patients in both the intima (Fig. 5A, *p* = 0.0192) and media (Fig. 5B, *p* < 0.0001). In contrast, a decrease was seen in late AMI + AI patients compared to early AMI + AI patients in the intima (Fig. 5A, *p* < 0.0001). All values are shown in Table 2. Examples of the tryptase staining is presented are Fig. 5D.

**Fig. 5:**
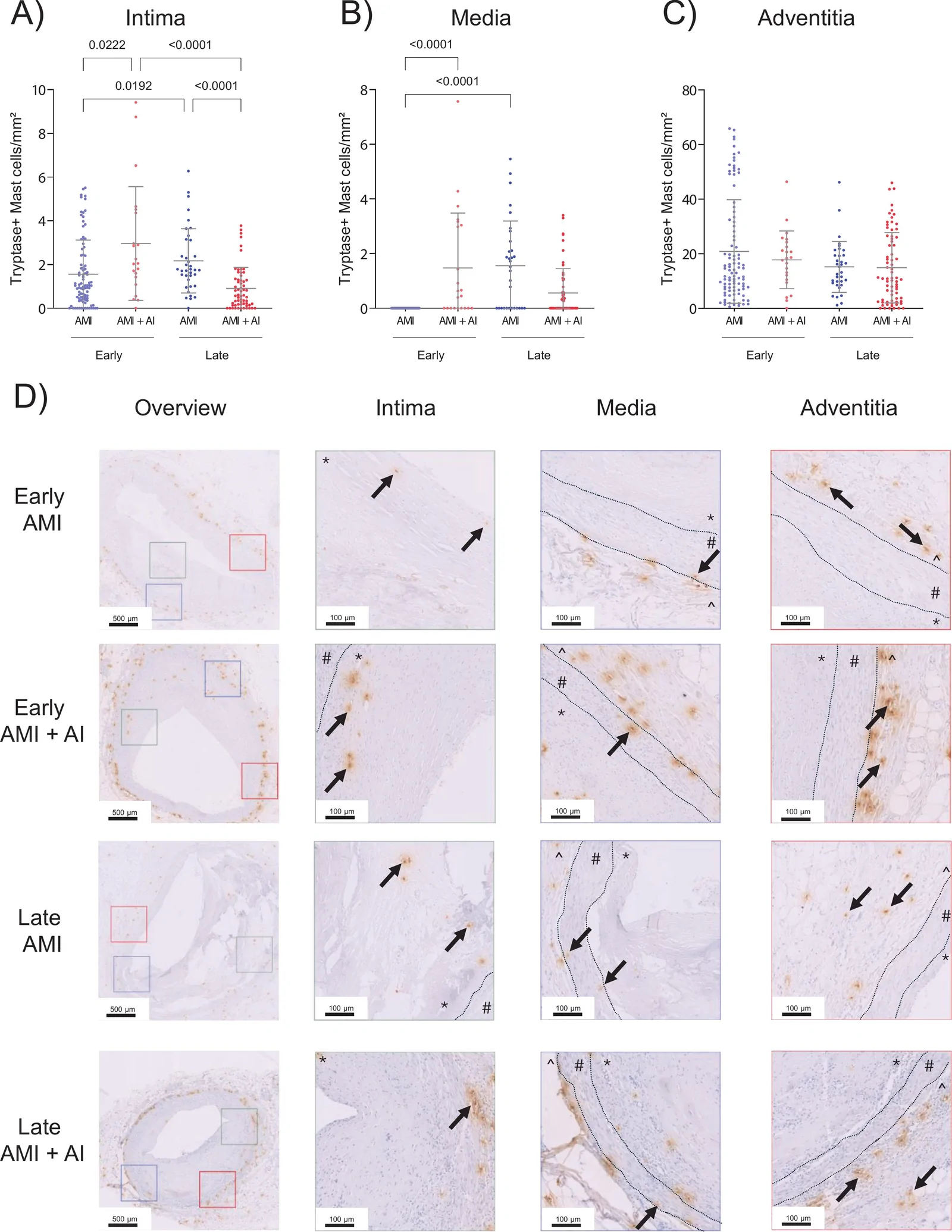
Tryptase + mast cell density per group. Tryptase+ mast cells/mm^2^ in either the intima, media or adventitia of the coronary arteries. Samples consist of atherosclerotic plaque cross sections. A: Density in mm^2^ of tryptase+ mast cells in the total intima area: Early AMI: *n* = 104, early AMI + AI: *n* = 23, late AMI: *n* = 37 and late AMI + AI: *n* = 74. Values were calculated via Kruskal-Wallis testing. B: Density in mm^2^ of tryptase+ mast cells in the total media area: Early AMI: *n* = 104, Early AMI + AI: *n* = 23, late AMI: *n* = 37 and late AMI + AI: *n* = 74. Values were calculated via Kruskal-Wallis testing. C: Density in mm^2^ of tryptase+ mast cells in the total adventitia area: Early AMI: *n* = 104, early AMI + AI: *n* = 23, late AMI: *n* = 37 and late AMI + AI: *n* = 74. Values were calculated via Kruskal-Wallis testing. D: MPO staining for neutrophils (brown colourization, arrow points to an example of positive staining) for all patient groups, with higher magnification of the intima, media and adventitia. If multiple layers are present in the higher magnification photograph, a dotted line divides the layers and an “I” (Intima), “M” (Media) or “A” (Adventitia) denotes the area of interest All samples are independent and were processed once. All graphs values were calculated via Kruskal-Wallis testing. Error bars represent mean and standard deviation, significant *p*-values (*p* < 0.05) are shown above the columns. (For interpretation of the references to colour in this figure legend, the reader is referred to the web version of this article.)

Of note, significantly increased mast cell numbers were found in the adventitia of non-infarct-related arteries (30.6 ± 28.0) compared to infarct-related arteries (17.61 ± 18.8) in early AMI patients (*p* = 0.0071). No other significant differences were found in the numbers of mast cells, or other inflammatory cells, in the walls of infarct- and non-infarct related coronary arteries.

## 4 Discussion

### 4.1 Summary

Given the short-term effects of systemic inflammation on cardiovascular event risk, we hypothesized that an acute episode of infection rapidly enhances intra-plaque inflammation and plaque instability. In addition, the presence of infection could contribute to the pro-inflammatory changes in the arterial wall that are evoked by recent AMI itself [15].

### 4.2 Mast cell increase and increased cardiovascular event risk

In AMI, we found that the presence of infection was associated with an increased mast cell density in the intima and media of coronary arteries. The density of other inflammatory cells, however, was not increased, nor was there a clear effect on histopathological signs of plaque instability. To our knowledge, we are the first to describe an increase of mast cells in coronary arteries as a result of infection. This increase is of potential interest since mast cells in the coronary arterial walls are linked to plaque destabilization [27], [28] and cardiovascular risk [28], [29]. Although mast cells are mostly found and studied in the adventitia, it has recently been suggested that mast cells in the intima and media of atherosclerotic coronary lesions may contribute to the onset of AMI through plaque-destabilizing or spasm-inducing properties [28], involving the release of mediators such as histamine, chymase, tryptase, growth factors, and pro-inflammatory cytokines [30], [31]. In several studies in atherosclerotic mouse models, inhibition of mast cells reduced plaque instability [32], [33], [34], [35], [36] whereas activation of mast cells had the opposite effect [33], [34], [37]. Moreover, the number of mast cells in human carotid atherosclerotic plaques was shown to be predictive for the occurrence of cardiovascular events, irrespective of their level of activation [29]. Previous studies have shown that comparable levels of mast cells are clinically relevant [38]. In addition, via release of vasoactive mediators, especially histamine, mast cells have been implicated in provoking coronary contraction or even spasms, which can reduce coronary flow, but also destabilize the plaque [39], [40], [41], [42]. Thus, our finding of enhanced mast cell density in the intima and media of AMI patients with acute infection may at least partially explain the increased cardiovascular event risk seen shortly after infection [4].

### 4.3 Effects of mid- to longer term changes on atherosclerotic plaques

Why only mast cells and not the other inflammatory cells are increased in the coronary arteries of patients with infection is not clear. This finding does, however, not fully exclude a role for activated CD45+ leukocytes and macrophage detection, because their activation may cause loss of epitopes and cells can become undetectable by MAC-3 and CD45-staining, respectively. Alternatively, and perhaps more likely, is that the CD45+ leukocytes density is a true representation of their role in early AMI occurring during acute infection. This may, for example, be explained by preferential recruitment of these cells in infected tissue elsewhere in the body. Interestingly, previously an increase of macrophages, T-cells and dendritic cells was found in the adventitia of AMI + AI patients [7], while we found no increase of macrophages in the intima. As we have demonstrated, the intima, media and adventitia have different amounts of inflammatory cells and cannot be easily compared to each other. In addition, our patients died between 2006 and 2013, ±15 to 20 years later than in the aforementioned study, potentially affecting medicine use (e.g. the anti-inflammatory statins).

With respect to the mid- to longer-term changes in coronary inflammation after AMI, our findings corroborate earlier murine studies showing that AMI itself contributes to more widespread plaque inflammation and instability [43]. Additionally it has been shown that an increase of leukocyte adhesion to platelets occurs in the culprit coronary artery, potentially resulting in reduced flow or an increased inflammatory environment [44]. An increase of leukocyte activation might play a role in coronary atherothrombosis in patients with STEMI [45]. The increase of leukocytes in late AMI patients might therefore affect plaque stability. Atherosclerotic plaque homeostasis is affected by macrophages, which can either be pro- or anti-inflammatory, causing generation of fibrotic tissue and calcification of necrotic lesions to stabilise plaques as a response to inflammation [46]. On the other hand, plaque stability can be decreased by breaking down of collagen in the fibrotic cap following secretion of metalloproteinases by macrophages [47]. The exact role of increased macrophages in late AMI and late AMI + AI can therefore not be determined in this study. Coronary plaque erosion can occur following activation of neutrophils [48]. Interestingly, inhibition of MPO, the neutrophil secreted enzyme that predicts rupture of atherosclerotic lesions, has been shown to reduce adverse cardiac events [49]. The increase of neutrophils in late stage AMI patients might therefore negatively affect plaque stability.

We did not find evidence that infection enhanced these post-AMI pro-inflammatory changes. An effect of post-AMI infection on plaque stability and subsequent cardiovascular event rate has been proposed [16], but our findings argue against a dominant role for plaque inflammation in this phenomenon.

### 4.4 Limitations

Important limitations have to be taken into consideration. Firstly, the total number of studied patients is low, particularly in subgroups. This small study is the first of its kind, but can, in view of the limited size of 5 patients in the early AMI with infection group compared to the early AMI group without infection with 12 patients, only be hypothesis-generating, although our main findings are still statistically significant. Ideally, more females would have been included, but more females with the correct inclusion criteria were not available in our biobank. In addition, the optimum time after AMI to study coronary arteries with respect to inflammatory activity is unknown. A maximum period of 6 h may not fully preclude effects of infarction event itself. For the patients that died between 6 h and 14 days after onset of AMI, infection could have been present either pre-AMI, or developed post-AMI at any given time. The high number of unknown STEMI or non-STEMI data of patients that did not receive an ECG can be considered a limitation. As STEMI status for over half of the patients is missing, we decided to not analyse and discuss differences between STEMI and non-STEMI patients. Changes might have occurred post-mortem that could affect the results of the staining (e.g. mast cell degranulation), although this would occur in all patients and would not explain differences between the different groups.

## 5 Conclusion

In conclusion, we found that the presence of infection increases mast cell numbers in the intima and media of coronary arteries, but does not seem to increase other inflammatory cells or increase plaque instability. Mast cell involvement may be a partial explanation for the increased cardiovascular event risk seen shortly after infection. We found no evidence that the presence of infection enhances the coronary pro-inflammatory changes that are evoked by recent AMI itself.

## CRediT authorship contribution statement

**M.D. Fiet:** Writing – review & editing, Writing – original draft, Visualization, Methodology, Investigation, Formal analysis, Conceptualization. **W.W. Fuijkschot:** Writing – original draft, Investigation, Formal analysis. **L. Woudstra:** Investigation, Data curation. **R. van der Linden:** Investigation. **D. Beskers:** Investigation. **I.P.A. Zethof:** Writing – review & editing. **L.F.H.J. Robbers:** Investigation. **S. Simsek:** Supervision. **P.A.J. Krijnen:** Writing – review & editing, Supervision. **H.W.M. Niessen:** Writing – review & editing, Supervision. **Y.M. Smulders:** Writing – review & editing.

## Statement of ethics

This study was approved by and performed according to the guidelines of the Ethics Committee of the AUMC, location VUmc, and conforms to the principles of the Declaration of Helsinki. Use of the leftover tissue for research purposes after the pathological examination has been completed in part of the patient contract in our hospital. Consent was not required for this study in accordance with the hospital guidelines of the Ethics Committee of the AUMC, location VUmc.

## Declaration of competing interest

The authors declare that they have no known competing financial interests or personal relationships that could have appeared to influence the work reported in this article.

## Data Availability

The data that support the findings of this study are available from the corresponding author upon request. The data are not publicly available due to privacy or ethical restrictions.

## Appendix A Supplementary data

Supplementary Fig. 1. Gram staining. A: Atherosclerotic plaque without presence of gram-positive bacteria. B: Lung tissue of a pneumonia patient, with presence of gram-positive bacteria (arrows). Supplementary Fig. 2. Isotype control for monoclonal antibodies. Coronary arteries stained with monoclonal primary antibodies (left) and their respective isotype control (right). Asterisks are used to demonstrate the same location of the tissue is used. Although the location is the same, no asterisk was placed for the tryptase staining due to the absence of identifying structures. A: CD45+ leukocytes (black arrow). B: CD68+ macrophages (black arrow). C: Tryptase + mast cell (black arrow). Supplementary Fig. 3. Negative control for polyclonal antibodies. Coronary arteries stained with polyclonal primary antibodies (left) and a negative control (right). The same location is illustrated per staining, with a zoom in the top right corner. A: MPO+ neutrophil (black arrow). B: GLUT-1+ cells (black arrow). Supplementary Fig. 4. Stenosis and intraplaque haemorrhage. A: NBT staining to determine LDH decolouration. B: Elastica von Gieson staining for determining stenosis. C: GLUT-1 staining for identifying fresh haemorrhage (brown colourization, black arrow). D: Perls staining for identifying old haemorrhage (magenta colourization, black arrow). The same plaque has been stained as indicated by the *. Supplementary Fig. 5. Plaque stability. EVG staining to demonstrate a stable and an unstable plaque. A: A stable plaque (thick cap, black arrow). B: An unstable plaque (thin cap, black arrow), with a zoom in to illustrate an inflammatory cell (black arrow).

Supplementary Table 1. Detailed AMI + AI patient characteristics. Overview of infarct duration, STEMI, infection, sepsis and comorbidity of AMI + AI patients. Supplementary Table 2. Detailed AMI patient characteristics. Overview of infarct duration, STEMI, and comorbidity of AMI patients.
