# Supplementary material for: Systemic inflammation increases mast cell numbers in coronary arteries in patients who died from myocardial infarction
Source: J Mol Cell Cardiol Plus. 2026 Sep 24;18:100870. doi: 10.1016/j.jmccpl.2026.100870 (PMC13625635; doi:10.1016/j.jmccpl.2026.100870)
Supplement: Supplementary file 1 [file mmc2.docx]

| Supplementary table 1: STEMI, infectious agents and comorbidities | | | | |
| --- | --- | --- | --- | --- |
| Patient | **Infarct duration** | **STEMI** | **Type of infection & sepsis** | **Comorbidities** |
| 1 | Early | Unknown | E. faecalis | DM2 |
| 2 | Early | Unknown | Unknown  Sepsis | Hypertension, CVA, DVT, LE, KF |
| 3 | Early | NSTEMI | Bacterial  Sepsis | Systemic sclerosis |
| 4 | Early | NSTEMI | Unknown | Cancer (prostate) |
| 5 | Early | NSTEMI | K. pneumoniae  Sepsis | DM2, cancer (prostate and colon), aortic prothesis, cholecystectomy |
| 6 | Late | NSTEMI | Unknown  Sepsis | AF |
| 7 | Late | NSTEMI | S. marcescens,  P. aeruginosa,  E. coli, Sepsis | CABG and mitral valve prothesis |
| 8 | Late | Unknown | Bacterial | Cancer (breast), hypertension, hypercholesterolemia, appendectomy, AF |
| 9 | Late | Unknown | Unknown | Aortic prothesis, DM2, hypercholesterolemia |
| 10 | Late | Unknown | E. faecalis | Gastrectomy, aortic valve stenosis |
| 11 | Late | NSTEMI | S. aureus, Gram+ | AMI (3 times) |
| 12 | Late | Unknown | Unknown  Sepsis | CABG, kidney failure, hypertension |
| 13 | Late | Unknown | E. cloacae,  S. maltophilia  Sepsis | Femoropopliteal bypass, COPD, DM2, hypertension, AMI, AF, KF |
| 14 | Late | NSTEMI | S. pyogenes  Sepsis | AMI (2 times) with CABG and HF, aortic aneurysm, KF, COPD |
| 15 | Late | Unknown | E. coli, P. putida,  C. albicans, Gram+  Sepsis | None |
| 16 | Late | NSTEMI | Unknown | None |
| 17 | Late | Unknown | C. tropicalis  Sepsis | AMI, vascular dementia, hypertension |

Abbreviations: STEMI: ST-elevated myocardial infarction, NSTEMI: Non-ST-elevated myocardial infarction, DM: Diabetes mellites type 2, CVA: Cerebral vascular accident, DVT: Deep vein thrombosis, LE: Lung embolism, KF: Kidney failure, AF: Atrial fibrillation, CABG: Coronary artery bypass grafting, AMI: Acute myocardial infarction; COPD: Chronic Obstructive Pulmonary Disease

| Supplementary table 2: STEMI and comorbidities | | | |
| --- | --- | --- | --- |
| Patient | **Infarct duration** | **STEMI** | **Comorbidities** |
| 1 | Early | Unknown | None |
| 2 | Early | NSTEMI | Lung carcinoma, AMI, HF, aortic valve stenosis |
| 3 | Early | STEMI | Cholelithiasis |
| 4 | Early | Unknown | AMI with CABG |
| 5 | Early | Unknown | AF, AMI |
| 6 | Early | Unknown | Dilated cardiomyopathy, alcoholic fibrosis (liver and heart) |
| 7 | Early | NSTEMI | Liver cirrhosis, aortic valve stenosis, LVH, otitis media |
| 8 | Early | Unknown | Trama capitis |
| 9 | Early | Unknown | None |
| 10 | Early | Unknown | COPD, AF |
| 11 | Early | Unknown | COPD, hypertension, DM2, systemic amyloidosis |
| 12 | Early | Unknown | None |
| 13 | Late | STEMI | DM2, sarcoidosis, AMI |
| 14 | Late | STEMI | DM2, cancer (prostate), alcohol abuse |
| 15 | Late | STEMI | None |
| 16 | Late | NSTEMI | Intracerebral haemorrhage |
| 17 | Late | STEMI | None |
| 18 | Late | Unknown | None |

Abbreviations: STEMI: ST-elevated myocardial infarction, NSTEMI: Non-ST-elevated myocardial infarction, AMI: Acute myocardial infarction, HF: Heart failure, CABG: Coronary artery bypass grafting, LVH: Left ventricular hypertrophy, COPD: Chronic obstructive pulmonary disease, AF: Atrial fibrillation; DM2: Diabetes mellites type 2
